# Level and Factors Associated with Participation in Population-Based Cancer Screening in Safranbolu District of Karabuk, Turkey

**Published:** 2020-04

**Authors:** Raziye ÖZDEMIR, Fatma TÜRKMEN ÇEVIK, Duygu KES, Merve KARACALI, Simge ÖZGÜNER

**Affiliations:** 1. Department of Occupational Health and Safety, Faculty of Health Sciences, Karabuk University, Karabuk, Turkey; 2. Safranbolu Community Health Center, Karabuk, Turkey; 3. Department of Nursing, Faculty of Health Sciences, Karabuk University, Karabuk, Turkey; 4. Eflani District Hospital, Karabuk, Turkey; 5. Karabuk Public Health Directorate, Karabuk, Turkey

**Keywords:** Population-based cancer screening, Cervix, Breast, Colorectal, Turkey

## Abstract

**Background:**

Cervix, breast and colorectal cancers are included in the national population-based screening (PBS) program in Turkey. This study aimed to assess participation in PBSs for these cancers and to identify factors associated with participation in screenings in Safranbolu district of Karabuk, Turkey in 2016–2017.

**Methods:**

In this cross-sectional study, separate studying groups for cervix, breast and colorectal cancers were identified, taking into account the target age range specified in the national screening standards. The sample size was determined to be 374 for cervical cancer, 371 for breast cancer and 373 for colorectal cancer in the Epi-Info StatCalc program with a prevalence of 50%, a 95% Confidence Interval (CI) and a 5% error margin. The results of the data collected through face-to-face interview using questionnaires were evaluated with Chi-square tests (*P*<0.05) and included in the binary logistic regression model.

**Results:**

Participation in PBS at least once between 2011 and 2016 years was 26.2% for cervical cancer, 27.6% for breast cancer and 31.6% for colorectal cancer, whereas the level of PBS or opportunistic screening at least once was 51.1%, 42.7% and 32.2%, respectively. A 2.9-fold increase in participation for the cervical cancer screening was associated with informing women about cervical cancer by the family physicians. Being married and living in the district center showed associations with a higher rate of participation for colorectal cancer screening.

**Conclusion:**

Participation in PBS was low for the 5.5-year period. More effort is needed to increase the effectiveness of the program.

## Introduction

Increased numbers of cancers due to changes in the demographic structure of communities and the spread of cancer risk factors have created a significant burden of disease in developing countries as well as in developed countries (1). In Turkey, cancer is the second-leading cause-of-death, following cardiovascular diseases (2), and about 148000 new cancer cases occurred in 2012 (3). The most common types of cancer are lung, prostate, bladder and colorectal for men and breast, thyroid, colorectal and stomach cancers for women (3).

WHO recommends that countries should implement cancer control programs, including surveillance, prevention, early detection, diagnosis/treatment and palliative care services to reduce the growing burden of cancer diseases. In Turkey, the first national cancer control program was designed in 2008 and updated in 2013 (4). The program focuses on two essential activities in the early detection of cancer. The first is to increase the level of individuals’ awareness of cancer symptoms and signs to detect them and to ensure that persons apply to the health facility.

The second is the detection of cancer in the asymptomatic period by population-based screening (PBS) programs for breast, cervix and colorectal cancers (4). The PBSs presented their national screening standards in Table 1 are available free of charge in the cancer early detection and screening centers. The provision of program integration of primary care workers is critical for the success of PBSs targeted to reach 70% of the eligible population for screening. In the program, family physicians (FPs) are given responsibility for inviting individuals to PBS, reporting screening results and directing persons who have a suspicious test result to the hospital for further examination and follow-up (4).

**Table 1:** Target population, screening tests and screening intervals for cancers with national screening standards

***Cancer type***	***Target population***	***Screening test***	***Screening interval***
Cervix	Women 30–65 yr of age	Pap-smear test	Once every five years
		HPV test^*^	Once every five years
Breast	Women 40–69 yr of age	Mammography	Once every two years
Colorectal	Women and men 50–70 yr of age	Faecal occult blood test	Once every two years
		Colonoscopy	Once every 10 years

* It was started on Aug 1, 2014.

PBS programs can reduce the burden of disease and mortality due to breast, cervical and colorectal cancers (1, 5–8). However, because of the lack of organized screening programs in developing countries, cancer is often diagnosed at a late stage leading to a decrease in the survival rate and an increase in disease burden and treatment costs (9, 10). It is important to know the level of functioning and performance of the current program which may help decision-makers for future planning and improvement of the services.

The aim of this study was to assess participation in PBSs for cervical, breast and colorectal cancers and to identify factors associated with participation in screenings between Jan 2011 and Jun 2016 in Safranbolu district of Karabuk.

## Methods

This cross-sectional study carried out in 2016–2017 years in Safranbolu district of Karabuk Province, northwest Turkey. Separate studying groups for cervix, breast and colorectal cancers were identified, taking into account the target age range specified in the national screening standards. The target population sizes determined by using data from the Turkish Statistical Institute (TURKSTAT) were 13836 for cervical cancer, 10530 for breast cancer and 13230 for colorectal cancer. The sample size was calculated to be 374 for cervical cancer, 371 for breast cancer and 373 for colorectal cancer, with a prevalence of 50% (unknown prevalence), 95% confidence intervals (CI) and a 5% error margin in the Epi-Info-7 StatCalc. In this manner, total study sample included 1118 individuals.

The study groups were distributed in proportion to the population living in the district center and the villages. The number of people over 18 yr of age residing in Safranbolu which has 20 neigh-bourhoods was obtained from TURKSTAT, and then seven clusters were formed based on population density. Sixty villages of Safranbolu were divided into another seven groups according to the responsibility areas of FPs. Seven neighbour-hoods and 20 villages were randomly selected from the groups formed. While collecting data from all neighbourhoods, however, not all villages could be visited for each cancer type due to logistical problems. Data were collected from 10 villages for cervical cancer, four villages for breast cancer and 18 villages for colorectal cancer. The interviews were conducted starting from a different street in the neighbourhoods and villages for each type of cancer, and one questionnaire for only one cancer type was filled out with an individual. Total 29 people refused to participate in the study. Finally, the questionnaires were completed with 1131 people.

The dependent variable was the level of participation in PBS for cervical, breast and colorectal cancers between Jan 2011 and Jun 2016. The level of PBS included invited and uninvited screenings. Invited screening indicated the proportion of individuals invited to PBS by the primary care workers to the total number of people in the study group, whereas uninvited screening expressed the proportion of individuals who had PBS by their own decision without invitation to the total number of people in the study group. Invitation coverage was calculated as the proportion of individuals in the eligible target age range receiving a screening invitation over the total number of the participants (11). The level of opportunistic screening was determined because it would also affect participation in PBS.

The independent variables were age, gender, place of residence, marital status, education level, employment status, husband’s education level, husband’s employment status, number of child, household income level, cancer patient in family, awareness of the PBS program and obtaining information about relevant cancer from FPs.

The data were collected through face-to-face interviews. Questionnaires, prepared by the researchers, included 47 items for cervix cancer, 50 items for breast cancer and 52 items for colorectal cancer. A pilot survey was conducted in a neighbourhood of Karabuk, where was outside of the study area, with 17 people.

The data were summarized as proportions and analyzed by the chi-square test and binary logistic regression. Variables with a statistically significant association on univariate analysis (*P*<0.05) were included in the logistic regression model.

The ethical approval and permission to conduct the study were obtained from Karabuk University Non-Interventional Clinical Research Ethics Board (No: 2015/10) and Karabuk Public Health Directorate respectively. In addition, verbal consent was obtained from all individuals for voluntary participation.

## Results

Overall, 1131 individuals, including 374 women for cervical cancer, 384 women for breast cancer and 373 women and men for colorectal cancer, were reached.

**Fig. 1:**
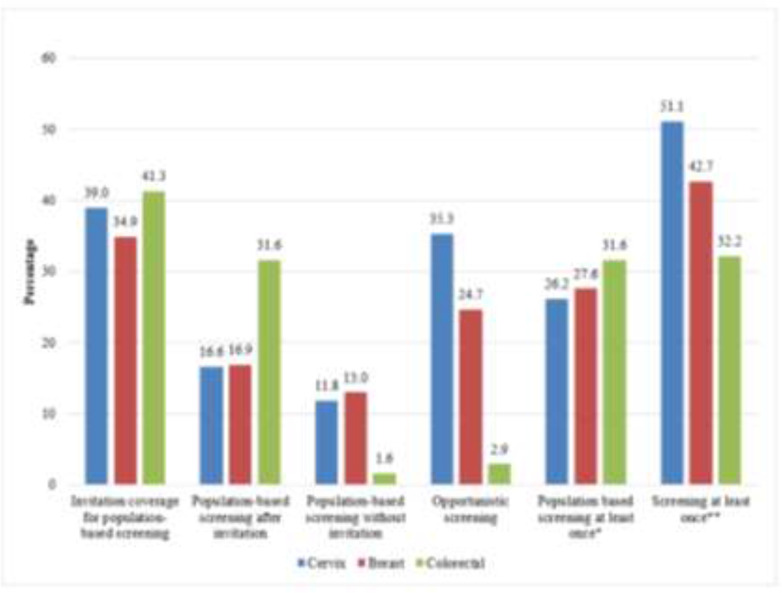
Screening activities for cervix, breast and colorectal cancers in Safranbolu, January 2011–June 2016 ^*^Population-based screening at least once with or without invitation ^**^Population-based or opportunistic screening at least once

For cervix cancer, the overall invitation coverage for PBS was 39.0%. The levels of invited and un-invited screenings were 16.6% and 11.8%, respectively. In this group, more than one-third of the women had undergone opportunistic screening; 26.2% of women had at least one PBS, and 51.0% of those had at least one screening (population-based or opportunistic) (Fig. 1). Two women’s test results were found suspicious after the PBSs. One of them applied to the hospital and was diagnosed with cervical intraepithelial neoplasia following further examination.

In the screening activity for breast cancer, opportunistic screening of 24.7% was followed by invited screening of 16.9% and uninvited screening of 13.0%. The proportion of women who had PBS at least once was 27.6% (Fig. 1). There were no women with a suspicious test result in this group.

Invitation coverage of PBS for colorectal cancer was 41.3%. The proportion of invited individuals to screening for colorectal cancer was higher than the others; however, the proportion of uninvited participants was as low as 1.6%. About one-third of the individuals had a screening at least once (Fig. 1). Ten individuals had a suspicious test result, however, only five of them applied to the hospital for further examination, and one person was diagnosed with in-situ cancer.

**Fig. 2:**
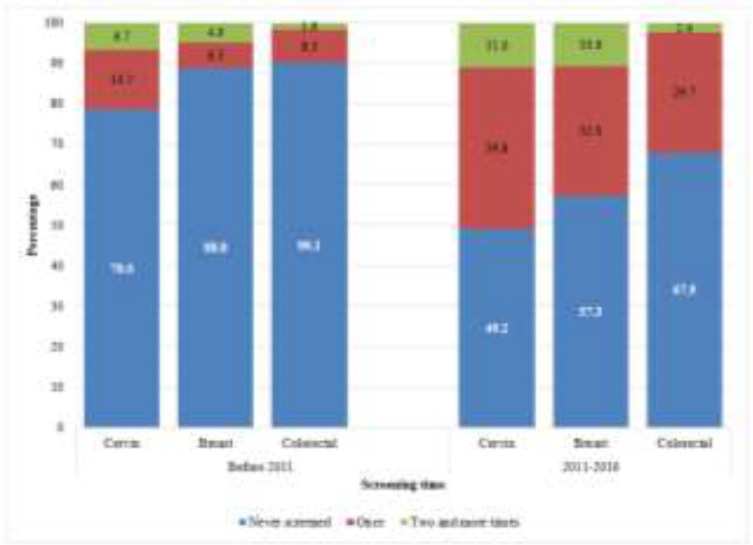
Distribution of screenings (population-based or opportunistic) for cervix, breast and colorectal cancers by screening time in Safranbolu

The levels of never-screened individuals were 78.6% for cervical cancer, 88.8% for breast cancer and 90.1% for colorectal cancer before 2011. These levels were 49.2% for cervical cancer, 57.3% for breast cancer and 67.0% for colorectal cancer between 2011 and 2016 (Fig. 2).

In Univariate analysis, participation in PBS for cervical cancer was higher in women who were aware of the screening program and informed about cervical cancer by FP (*P*<0.001). In logistic regression analysis, unawareness of cervical cancer screening program was decreased participation in screening by 2.8 times (*P*= 0.005). Participation in screening in the women not informed about cervical cancer by FP was low by 2.9 times (*P*<0.001). Other variables did not affect the participation rate (*P*>0.05) (Table 2).

**Table 2:** The association between participation in population-based cervical cancer screening and independent variables (screened= 0, non-screened= 1)

***Variable***	***Screened***	***Not screened***	***Univariate analysis***		***Logistic regression analysis***		
	n (%^*^)	n (%^*^)	***Chi-square***	**P**	***OR***	***95% CI***	**P**
Age group							
30–47	44 (25.3)	130 (74.7)	0.141	0.707	-		
48–65	54 (27.0)	146 (73.0)					
Place of residence							
District centre	83 (27.4)	220 (72.6)	1.168	0.280			
Village	15 (21.1)	56 (78.9)					
Marital status							
Married	87 (27.0)	235 (73.0)	0.796	0.372			
Unmarried^**^	11 (21.2)	41 (78.8)					
The number of child							
None	15 (23.1)	50 (76.9)	1.607	0.448			
1–3	44 (29.7)	104 (70.3)					
≥4	39 (24.2)	122 (75.8)					
Education level							
Primary school and below	75 (26.6)	207 (73.4)	0.091	0.762			
Secondary school or above	23 (25.0)	69 (75.0)					
Employment status							
Employed	12 (24.5)	37 (75.5)	0.086	0.770			
Unemployed	86 (26.5)	239 (73.5)					
Husband’s education level							
Primary school and below	39 (23.8)	125 (76.2)	1.425	0.261			
Secondary school or above	48 (29.6)	114 (70.4)					
Husband’s employment status							
Employed	53 (27.3)	141 (72.7)					
Unemployed	2 (16.7)	10 (83.3)	0.656	0.720			
Retired	32 (26.9)	87 (73.1)					
Monthly household income							
≤1500 liras (450 EUR)	52 (28.0)	134 (72.0)					
>1500 liras (450 EUR)	46 (24.5)	142 (75.5)	0.589	0.443			
Cancer patient in the family							
Yes	32 (24.6)	98 (75.5)					
No	66 (27.0)	178 (73.0)	0.26	0.610			
Awareness of cervical cancer screening program							
Yes (ref)	86 (34.1)	166 (65.9)			1.0		
No	12 (9.8)	110 (90.2)	25.083	0.000	2.8	[1.4–5.7]	0.005
Informed about cervical cancer by the family physician							
Yes (ref)	76 (38.2)	123 (61.8)			1.0		
No	22 (12.6)	153 (87.4)	31.606	0.000	2.9	[1.6–5.2]	0.000
Total	98 (26.2)	276 (73.8)					

ref: Reference variable

* Row percentage

** Single, widowed, divorced and separated

The awareness of the screening program was the main factor affecting participation in breast cancer screening (OR= 2.8, *P*= 0.010). Also, the rate of participation was decreased by 4.1 times in women who had not undergone clinical examination by the FP (95%CI 1.0–17.3, *P*= 0.051) (Table 3).

**Table 3:** The association between participation in population-based breast cancer screening and independent variables (screened= 0, non-screened= 1)

**Variable**	***Screened***	***Not screened***	***Univariate analysis***		***Logistic regression analysis***		
	n% ^*^	n% ^*^	***Chi-square***	**P**	***OR***	***95% CI***	**P**
Age group							
40–49	50 (27.9)	129 (72.1)	0.112	0.945			
50–59	37 (28.8)	95 (72.0)					
60–69	19 (26.0)	54 (74.0)					
Place of residence							
District centre	82 (27.9)	212 (72.1)	0.052	0.820			
Village	24 (26.7)	66 (73.3)					
Marital Status							
Married	86 (26.5)	239 (73.5)	1.382	0.268			
Unmarried ^**^	20 (33.9)	39 (66.1)					
The number of child							
None	5 (41.7)	7 (58.3)	3.431	0.18			
1–3	80 (25.6)	232 (74.4)					
≥4	21 (35.0)	39 (65.0)					
Education level							
Primary school and below	73 (25.0)	219 (75.0)	4.136	0.042	1.6	[0.9–2.6]	0.099
Secondary school or above (ref)	33 (35.9)	59 (64.1)			1.0		
Employment status							
Employed	16 (31.4)	35 (68.6)	0.418	0.518			
Unemployed	90 (27.0)	243 (73.0)					
Husband’s education level							
Primary school and below	38 (22.2)	133 (77.8)	3.509	0.061			
Secondary school or above	51 (31.3)	112 (68.7)					
Husband’s employment status							
Employed	54 (28.0)	139 (72.0)	1.911	0.385			
Unemployed	1 (9.1)	10 (90.9)					
Retired	34 (26.4)	95 (73.6)					
Monthly household income							
≤1500 liras (450 EUR)	57 (30.5)	130 (69.5)	1.51	0.219			
>1500 liras (450 EUR)	49 (24.9)	148 (75.1)					
Cancer patient in the family							
Yes	39 (27.9)	101 (72.1)	0.007	0.933			
No	67 (27.5)	177 (72.5)					
Awareness of breast cancer screening program							
Yes (ref)	80 (32.9)	163 (67.1)	9.364	0.002	1.0		
No	26 (18.4)	115 (81.6)			2.0	[1.2–3.3]	0.010
Informed about breast cancer by the family physician							
Yes (ref)	62 (33.0)	126 (67.0)	5.324	0.021	1.0		
No	44 (22.4)	152 (77.6)			1.6	[1.0–2.5]	0.058
Doing breast self-exam							
Once a month	50 (30.1)	116 (69.9)	1.005	0.605			
Once every 3 months and longer	32 (26.4)	89 (73.6)					
Non-practicing	24 (24.7)	73 (75.3)					
Clinical examination by family physician							
Yes (ref)	6 (66.7)	3 (33.3)	7.037	0.008	1.0		
No	100 (26.7)	275 (73.3)			4.1	[1.0–17.3]	0.051
Total	106 (27.6)	278 (72.4)					

ref: Reference variable

* Row percentage

** Single, widowed, divorced and separated

Being unmarried (OR= 4.1) and living in the village (OR= 2.4) showed independent associations with a lower participation rate for colorectal cancer screening Awareness of colorectal cancer screening program increased participation in screening (*P*<0.001). There was no participant in the screening among the individuals not given health education about colorectal cancer by the FP (Table 4).

**Table 4:** The association between participation in population-based colorectal cancer screening and independent variables (screened= 0, non-screened= 1)

	***Screened***	***Not screened***	***Univariate analysis***		***Logistic regression analysis***		
Variable	n%^*^	n%^*^	Chi-square	P	OR	95% CI	P
Age group(yr)							
50–59	60 (33.9)	117 (66.1)	0.798	0.372			
60–70	58 (29.6)	138 (70.4)					
Gender							
Female	65 (34.6)	123 (65.4)	1.514	0.219			
Male	53 (28.6)	132 (71.4)					
Place of residence							
District centre (ref)	99 (36.0)	176 (64.0)	9.219	0.002	1.0		
Village	19 (19.4)	79 (80.6)			2.4	[1.3–4.2]	0.003
Marital Status							
Married (ref)	113 (34.5)	215 (65.5)	9.967	0.001	1.0		
Unmarried ^**^	5 (11.1)	40 (88.9)			4.1	[1.5–10.8]	0.005
The number of child							
None	6 (42.9)	8 (57.1)	5.497	0.064			
1–3	93 (34.2)	179 (65.8)					
4+	19 (21.8)	68 (78.2)					
Education level							
Primary school and below	88 (30.1)	204 (69.9)	1.396	0.237			
Secondary school or above	30 (37.0)	51 (63.0)					
Employment status							
Employed	6 (19.49	25 (80.6)	3.209	0.201			
Unemployed	54 (30.5)	123 (69.5)					
Retired	58 (35.2)	107 (64.8)					
Monthly household income							
≤1500 liras (450 EUR)	55 (26.7)	151 (73.3)	5.184	0.023	1.4	[0.9–2.1]	0.200
>1500 liras (450 EUR) (ref)	63 (37.7)	104 (62.3)			1.0		
Cancer patient in the family							
Yes	41 (32.3)	86 (67.7)	0.037	0.847			
No	77 (31.3)	169 (68.7)					
Awareness of colorectal cancer screening program							
Yes	116 (59.2)	80 (40.8)	144.936	0.000	^a^		
No	2 (1.1)	175 (98.9)					
Informed about colorectal cancer by the family physician ^**^							
Yes	118 (69.0)	53 (31.0)	-	-	^b^		
No	0 (−)	202 (100.0)					
Total	118 (31.6)	255 (68.4)					

ref: Reference variable

* Row percentage

** Single, widowed, divorced and separated

a The analysis was not conducted, because only two individuals were screened among the people unaware of the screening program.

b The analysis was not conducted, because no one was screened among the uninformed people.

## Discussion

This study presents the level and factors associated with participation in PBSs for cervical, breast and colorectal cancers and provides important information on the performance of early-detection services. The study showed that the proportion of individuals screened by PBSs during the 5.5-year study period was very low in Safranbolu. Especially most of cervical and breast cancer screenings were opportunistic. The awareness of the screening program and screening recommendations made by the FPs were the strong determinants of participation in the screenings.

Key indicators reflecting the effectiveness of PBS programs and their acceptability by the community are invitation coverage and participation in screening after invitation (11). This study indicated that these levels were too low. Participation in PBSs is high in developed countries with strong primary care organizations and organized programs (12–14). Organised programs for breast, cervical and colorectal cancers are being conducted successfully in the majority of member states of the European Union. In the member states of the European Union, invitation coverage and participation rates were determined to be 79% and 60% for breast cancer, 59% and 51% for cervical cancer and 33% and 14% for colorectal cancer screening, respectively (11).

This study showed that a large part of breast and cervical cancer screenings were performed through opportunistic screenings. Compared to PBS, opportunistic screening has significant limitations. First, opportunistic screenings are available to a limited number of people, and socioeconomically disadvantaged individuals generally cannot access screening services. Women with higher education, of higher social class, living in urban areas and regularly visiting a gynaecologist were more likely to participate in cervical cancer screening than those with no habit of visiting a gynaecologist, of low social class, less educated, disabled, unemployed and with high cervical cancer risk (14). Another limitation of opportunistic screening is that it can cost more than PBS and lead to overuse of screening services (14–16). In Hong Kong, opportunistic screening for cervical cytology had a 40% reduction in a lifetime cervical cancer risk compared to no screening, whereas PBS with conventional or liquid-based cytology once every three, four and five years reduced it by 83%–93% (17).

The World Cancer Declaration Progress Report stated that the rate of cervical and breast cancer screening in Turkey had reached 80% and 30%, respectively (18). In this study, the screening level at least once through PBS or opportunistic screening was 51.1% for cervical cancer, 42.7% for breast cancer and 32.2% for colorectal cancer for the 5.5-year period. Considering that the screening for breast and colorectal cancers is done every two years, the screening levels drop to 10.6% for breast and 2.4% for colorectal. The level of screening in Safranbolu is below the national level and show an inability to maintain the continuity of the screenings.

This study showed that awareness of the Ministry of Health’s free screening program and to be informed by FPs had a significant impact on individuals’ screening behaviour. The reason for the more successful PBS programs in Europe is that primary care physicians play a supporting, informative and facilitating role for people to participate in these programs (19, 20). However, in this study, low invitation coverage and low health education levels point out the integration problems of FPs into the screening program. Effective health education ensures continuity of screening by increasing awareness and knowledge by individuals. Considering that even in well-organized PBS programs, participation rate in screening has not exceeded 80% (14), the importance of health education for newly launched screening programs can be better understood. In China, the physician’s suggestion of testing was the most effective way to increase the level of colorectal cancer screening, which was low due to the lack of information and socioeconomic and individual obstacles (21).

The success of PBS programs is not only due to the high screening coverage but also to conduct further examinations of suspicious test results and by providing treatment and monitoring of lesion-detected cases (22). In this study, half of the persons with suspicious outcomes after screening for cervical and colorectal cancer screenings did not undergo further examination to confirm their test results. The benefits of screenings will be limited unless the follow-up of individuals after screening is performed.

This study showed that individuals living in villages and unmarried had fewer FOBTs than individuals living in district center and married. A study in the Basque Country in Spain indicated that gender and socioeconomic differences affected the rate of participation in colorectal cancer screening and the frequency of lesions found in participants (23). In Japan, screening rates for colon, stomach and lung cancers had been reported to increase with marriage, living in a non-metropolitan area, having a high income and employment in a large-scale workplace (24).

Although this study has some limitations such as being generalized to a region and having a limited number of independent variables, it highlighted important issues related to the performance of PBSs and the factors affecting participation in PBS. The number of individuals participating in PBS in Safranbolu was far from the target of screening 70% of the eligible population. A large part of breast and cervical cancer screenings constituted opportunistic screenings. The study also highlighted the crucial role of FPs in the provision of preventive services and screenings for the cancers covered by PBS program.

## Conclusion

The rate of PBS application between 2011 and 2016 years was very low. There is a need for effective health education activities involving primary care and other levels of healthcare organizations to improve the screening program. Increasing public knowledge related to PBSs’ preventive role may be an effective way to enhance participation in the program. Comprehensive studies are also needed to identify the service providers and healthcare system-related barriers to participation in the screening program.

## Ethical considerations

Ethical issues (Including plagiarism, informed consent, misconduct, data fabrication and/or falsification, double publication and/or submission, redundancy, etc.) have been completely observed by the authors.

## References

[1] World Health Organization (2002). National cancer control programmes: policies and managerial guidelines. 2nd ed. World Health Organization Geneva.

[2] Turkish Ministry of Health Refik Saydam Hygiene Center Presidency School of Public Health Directorate (2006). Turkey Burden of Disease Study 2004, Unuvar N Mollahaliloğlu S Yardım NEds. Ankara, Turkey.

[3] Ferlay J Soerjomataram I Ervik M (2013). GLOBOCAN 2012 v1.0, Cancer Incidence and Mortality Worldwide: IARC CancerBase No. 11, International Agency for Research on Cancer, Lyon, France Available from: http://globocan.iarc.fr/Default.aspx

[4] Ministry of Health Turkey Public Health Institution Cancer Control Department (2016). Turkey Cancer Control Program Ankara Available from: https://www.iccp-portal.org/system/files/plans/Turkiye_Kanser_Kontrol_Program_English.pdf

[5] McCoy CB Pereyra M Metsch LR (2004). A community-based breast cancer screening program for medically underserved women: Its effect on disease stage at diagnosis and on hazard of death. Rev Panam Salud Publica, 15(3):160–7. 15096288 10.1590/s1020-49892004000300004

[6] Mandel JS Bond JH Church TR (1993). Reducing mortality from colorectal cancer by screening for fecal occult blood. NEJM, 328:1365–71. 8474513 10.1056/NEJM199305133281901

[7] Denny L Quinn M Sankaranarayanan R (2006). Screening for cervical cancer in developing countries. Vaccine, 24 Suppl 3: S3/71–7. 10.1016/j.vaccine.2006.05.121 16950020

[8] Landy R Pesola F Castañón A (2016). Impact of cervical screening on cervical cancer mortality: estimation using stage-specific results from a nested case–control study. Br J Cancer, 115(9):1140–46. 27632376 10.1038/bjc.2016.290 PMC5117785

[9] Sherris J Herdman C Elias C (2001). Beyond our borders cervical cancer in the developing world. West J Med, 175:231–33. 11577044 10.1136/ewjm.175.4.231 PMC1071564

[10] Singh GK Azuine RE Siahpush M (2012). Global ınequalities in cervical cancer incidence and mortality are linked to deprivation, low socioeconomic status, and human development. Int J MCH AIDS, 1(1):17–30. 27621956 10.21106/ijma.12 PMC4948158

[11] European Commission (2017). Cancer Screening in the European Union (2017) Report on the implementation of the Council Recommendation on cancer screening. Available from: https://ec.europa.eu/health/sites/health/files/major_chronic_diseases/docs/2017_cancerscreening_2ndreportimplementation_en.pdf

[12] Gakidou E Nordhagen S Obermeyer Z (2008). Coverage of cervical cancer screening in 57 countries: Low average levels and large inequalities. PLoS Med, 5(6):e132. 18563963 10.1371/journal.pmed.0050132 PMC2429949

[13] Klabunde C Blom J Bulliard JL (2015). Participation rates for organized colorectal cancer screening programmes: an international comparison. J Med Screen, 22:119–126. 25967088 10.1177/0969141315584694

[14] Döbrőssy L Kovács A Budai A (2015). Inequalities in cervical screening practices in Europe. Diversity and Equality in Health and Care, 12(2): 48–53.

[15] Nieminen P Kallio M Anttila A (1999). Hakama M. Organised versus spontaneous pap-smear screening for cervical cancer, a casecontrol study. Int J Cancer, 83:55–8. 10449608 10.1002/(sici)1097-0215(19990924)83:1<55::aid-ijc11>3.0.co;2-u

[16] Adab P McGhee SM Yanova J (2004). Effectiveness and efficiency of opportunistic cervical cancer screening: comparison with organized screening. Med Care, 42(6):600–9. 15167328 10.1097/01.mlr.0000128007.04494.29

[17] Kim JJ Leung GM Woo PP (2004). Cost-effectiveness of organized versus opportunistic cervical cytology screening in Hong Kong. J Public Health (Oxf), 26(2):130–7. 15284314 10.1093/pubmed/fdh138

[18] Union for International Cancer Control (UICC) (2016). World Cancer Declaration Progress Report 2016, Graham K Hakam Y Kaza N Mikhail M Morton Doherty R Tasker R Torode J Von der Muhll V eds. p.85 Available from: https://www.uicc.org/wcd-report

[19] Triantafillidis JK Vagianos C Gikas A (2017). Screening for colorectal cancer: the role of the primary care physician. Eur J Gastroenterol Hepatol, 29:e1–e7. 27676092 10.1097/MEG.0000000000000759 PMC5134820

[20] Khalid-de Bakker C Jonkers D Smits K (2011). Participation in colorectal cancer screening trials after first-time invitation: a systematic review. Endoscopy, 43:1059–86. 22135196 10.1055/s-0031-1291430

[21] Sung JJ Choi SY Chan FK (2008). Obstacles to colorectal cancer screening in Chinese: a study based on the health belief model. Am J Gastroenterol, 103:974–81. 18047545 10.1111/j.1572-0241.2007.01649.x

[22] Anttila A Lönnberg S Ponti A (2015). Towards better implementation of cancer screening in Europe through improved monitoring and evaluation and greater engagement of cancer registries. Eur J Cancer, 51(2):241–51. 25483785 10.1016/j.ejca.2014.10.022

[23] Hurtado JL Bacigalupe A Calvo M (2015). Social inequalities in a population-based colorectal cancer screening programme in the Basque Country. BMC Public Health, 15:1021. 26438240 10.1186/s12889-015-2370-5 PMC4594998

[24] Fukuda Y Nakamura K Takano T (2007). Socioeconomic status and cancer screening in Japanese males: Large inequality in middle-aged and urban residents. Environ Health Prev Med, 12(2):90–6. 21431825 10.1007/BF02898155 PMC2723645

